# The influence of a CYP1A2 polymorphism on the ergogenic effects of caffeine

**DOI:** 10.1186/1550-2783-9-7

**Published:** 2012-03-15

**Authors:** Christopher J Womack, Michael J Saunders, Marta K Bechtel, David J Bolton, Michael Martin, Nicholas D Luden, Wade Dunham, Melyssa Hancock

**Affiliations:** 1 Human Performance Laboratory, Department of Kinesiology, James Madison University, 261 Bluestone Drive, MSC 230, Harrisonburg, VA 22801, USA; 2 Department of Biology, James Madison University, Harrisonburg, VA 22801, USA

**Keywords:** Genetics, Cycling, Caffeine, Endurance performance

## Abstract

**Background:**

Although caffeine supplementation improves performance, the ergogenic effect is variable. The cause(s) of this variability are unknown. A (C/A) single nucleotide polymorphism at intron 1 of the cytochrome P450 (CYP1A2) gene influences caffeine metabolism and clinical outcomes from caffeine ingestion. The purpose of this study was to determine if this polymorphism influences the ergogenic effect of caffeine supplementation.

**Methods:**

Thirty-five trained male cyclists (age = 25.0 ± 7.3 yrs, height = 178.2 ± 8.8 cm, weight = 74.3 ± 8.8 kg, VO_2_max = 59.35 ± 9.72 ml·kg^-1^·min^-1^) participated in two computer-simulated 40-kilometer time trials on a cycle ergometer. Each test was performed one hour following ingestion of 6 mg·kg^-1 ^of anhydrous caffeine or a placebo administered in double-blind fashion. DNA was obtained from whole blood samples and genotyped using restriction fragment length polymorphism-polymerase chain reaction. Participants were classified as AA homozygotes (N = 16) or C allele carriers (N = 19). The effects of treatment (caffeine, placebo) and the treatment × genotype interaction were assessed using Repeated Measures Analysis of Variance.

**Results:**

Caffeine supplementation reduced 40 kilometer time by a greater (*p *< 0.05) magnitude in AA homozygotes (4.9%; caffeine = 72.4 ± 4.2 min, placebo = 76.1 ± 5.8 min) as compared to C allele carriers (1.8%; caffeine = 70.9 ± 4.3 min, placebo = 72.2 ± 4.2 min).

**Conclusions:**

Results suggest that individuals homozygous for the A allele of this polymorphism may have a larger ergogenic effect following caffeine ingestion.

## Background

Prior studies have established the ergogenic benefits of caffeine for both high-intensity short-duration performances [1-3], as well as endurance performance [4-6]. However, based on two studies that have reported individual data [3,6], approximately 30% of participants derive no ergogenic effects from caffeine ingestion. Doherty et al. [3] observed that four out of 14 subjects had no appreciable change in time to fatigue during running at a supramaximal workload following ingesting of caffeine. Meyers and Cafarelli [6] investigated the effects of acute caffeine supplementation on time to fatigue during repetitive quadriceps contractions. Three out of the 10 study participants did not respond to the caffeine or exhibited a worse performance under caffeine versus the placebo. Furthermore, not all studies report a significant ergogenic effect [7-9]. Beck et al. [7] did not observe any effect of caffeine on either maximal bench press strength or time to fatigue at 85% VO_2_max. Jacobson et al. [8] observed that caffeine had no additive effect on time trial performance when administered with pre-exercise carbohydrate or fat feedings. Finally, caffeine had no effect on peak power output or total work in a short-duration maximal cycling test [9]. Thus, the ergogenic effect of caffeine, while evident, is highly variable. The cause(s) of this variability across individuals remains unclear, and it is unknown if any of this variance is accounted for by genetic polymorphisms.

Cytochrome P450 is a hepatic enzyme that is a key component of caffeine metabolism. A (C/A) single nucleotide polymorphism at intron 1 of the cytochrome P450 gene influences the inducibility of this enzyme, with the C variant affecting a slower caffeine metabolism following caffeine ingestion in smokers [10]. This polymorphism has clinical importance, as caffeine increases risk for cardiovascular disease in individuals who possess the C variant, but not in individuals homozygous for the A variant [11,12], presumably due to a slower caffeine clearance in the former group. In contrast, Hallstrom et al. [13] observed that coffee consumption contributes to low bone mineral density in individuals homozygous for the A variant, and not those who possess the C allele. Therefore, this genetic polymorphism is a potential candidate to explain variability of ergogenic response to caffeine supplementation. The purpose of the present study was to determine if this specific CYP1A2 polymorphism influences the ergogenic effect of caffeine supplementation in trained cyclists.

## Methods

### Subjects

A total of 36 male recreationally competitive cyclists participated in the present study. One of these participants was excluded from the study post-hoc, as their cycling performance differed by more than two standard deviations from the mean value of the group. Therefore, 35 cyclists (age = 25.0 ± 7.3 yrs, height = 178.2 ± 8.8 cm, weight = 74.3 ± 8.8 kg, VO_2_max = 59.35 ± 9.72 ml·kg^-1^·min^-1^) were used for data analysis. Written informed consent was obtained from all participants prior to participation and the study and consent form were approved by the James Madison University Institutional Review Board. Habitual caffeine intake was self-reported by participants. Briefly, participants were asked for their average weekly intake of coffee, tea, soda, chocolate, and other caffeinated beverages. Typical milligram doses [14] were assigned to each and an approximate daily intake was obtained. Based on previous criteria [15], participants were then characterized as having low (0-150 mg·day^-1^), moderate (151-300 mg·day^-1^) and high (> 300 mg·day^-1^) caffeine intake.

### Maximal exercise test

Cyclists began the test at a work rate of 150 W on an electrically braked cycle ergometer, with load increases of 20 W each minute until volitional exhaustion. Maximal oxygen uptake (VO_2_max) was defined as the highest 1-minute oxygen value obtained during the test. Oxygen uptake (VO_2_) was monitored continuously via a Sensormedics Vmax (Yorba Linda, CA) metabolic measurement system calibrated in advance of all tests. Heart rate was monitored throughout the test using a Polar Heart Rate Monitor (Lake Success, NY).

### 40-kilometer time trial

Time trials were performed on two separate occasions. All testing was done in the morning following a 12-hour fast and at least 24 hours after any caffeine ingestion. Subjects were instructed to maintain their training and not increase or decrease their volume or intensity over the course of the study. One hour prior to testing, cyclists ingested capsules containing either 6 mg of anhydrous caffeine per kilogram body weight or white flour (placebo) randomly administered in double-blind fashion. Time trials were performed on an indoor cycle trainer (Velotron; Racermate, Seattle, WA) on a computer-simulated course. The course consisted of eight laps of a flat, five-kilometer loop. Cyclists were free to self-select the resistance by changing gears during the test and were allowed to track distance completed on the course via a video display. However, they were blinded to their time, speed, and power output during the trials. Water was available for the cyclists to ingest ad libitum. Oxygen uptake and respiratory exchange ratio (RER) were obtained and averaged over the last two minutes of each lap. Heart rate and Ratings of Perceived Exertion (RPE; using the original 6-20 Borg scale) were obtained at the end of each lap.

### Genotyping

Investigators were blinded to genotype until the subject completed the study. Furthermore, all genotyping was performed by an investigator not involved with the performance testing. DNA was obtained from whole blood samples via a QiaAmp mini-blood kit (Qiagen Inc.; Valencia, CA). Each blood sample was obtained prior to one of the cycling trials. Genotyping was performed using restriction fragment length polymorphism-polymerase chain reaction (RFLP-PCR), as previously described [12]. Briefly, DNA was PCR amplified using the HotStar DNA Polymerase Kit (Qiagen) with the forward primer (5'-CAACCCTGCCAATCTCAAGCAC-3') and reverse primer (5'-AGAAGCTCTGTGGCCGAGAAGG-3') to generate a 920 bp fragment of the CYP1A2 gene. PCR conditions consisted of an initial denaturation at 95°C for 5 minutes, followed by 39 cycles at 94°C for 15 seconds, 64.5°C for 1 minute, and 72°C for 1 minute, with a final elongation step of 72°C for 10 minutes. One half of each PCR product was digested using the restriction enzyme *ApaI *(New England Biolabs, Ipswich, MA) as per manufacturer's instructions. Digested and undigested PCR products were evaluated in parallel via electrophoresis in a 2% agarose gel stained with ethidium bromide, and DNA bands were visualized by UV light. The presence of a 920 bp fragment following *ApaI *digestion identified the A/A genotype, while the presence of 709 bp and 211 bp fragments following *ApaI *digestion identified the C/C genotype. Caffeine metabolism is similar between heterozygotes and CC homozygotes [10]. Therefore, similar to previous studies [11,12], cyclists were grouped as AA homozygotes and C allele carriers; the latter group including both heterozygotes and CC homozygotes.

### Statistical analyses

Descriptive data (height, weight, age, VO_2_max, caffeine intake) were compared between groups using independent t-tests. The frequency of low, moderate and high caffeine intake in the two genetic groups was compared using a Chi-Squared analysis. Potential differences in 40-km time, average VO_2_, HR, RER and RPE were assessed using repeated measures analysis of variance (RMANOVA) with treatment as a within-subjects factor and genotype as a between-subjects factor. For all RMANOVA procedures, post-hoc tests were performed using independent and dependent t-tests with a Bonferroni correction such that *P *< 0.025 was required for significance.

## Results

Out of the 35 participants analyzed, 16 (46%) were homozygous for the A variant and 19 (54%) were C allele carriers. This distribution is very similar to previously reported studies [10-12,15]. Descriptive characteristics of the two genotype groups are shown in Table 1. There were no significant differences (*p *> 0.05) between the two groups for height, weight, age, VO_2_max, or caffeine intake. In AA homozygotes, 12 out of the 16 participants were categorized as having low caffeine intake, three as moderate and one as high. Sixteen out of the 19 C allele carriers had low intake, one had moderate intake, with two characterized as having high intake. There was no difference in the distribution of low, moderate and high caffeine use between the two groups (*p *= 0.44).

**Table 1:** Descriptive data for AA homozygotes and C allele carriers

	A/A (n = 16)	C (n = 19)
Height (cm)	179.1 ± 10.6	178.0 ± 7.1

Weight (kg)	74.3 ± 12.5	73.7 ± 12.2

Age	24.0 ± 6.9	26.1 ± 7.8

VO_2_max (L·min^-1^)	4.30 ± 0.45	4.31 ± 0.58

VO_2_max (ml·kg^-1^·min^-1^)	59.04 ± 9.29	59.61 ± 10.31

Caffeine intake (mg per day)	85.71 ± 106.49	86.62 ± 145.40

**Figure 1:**
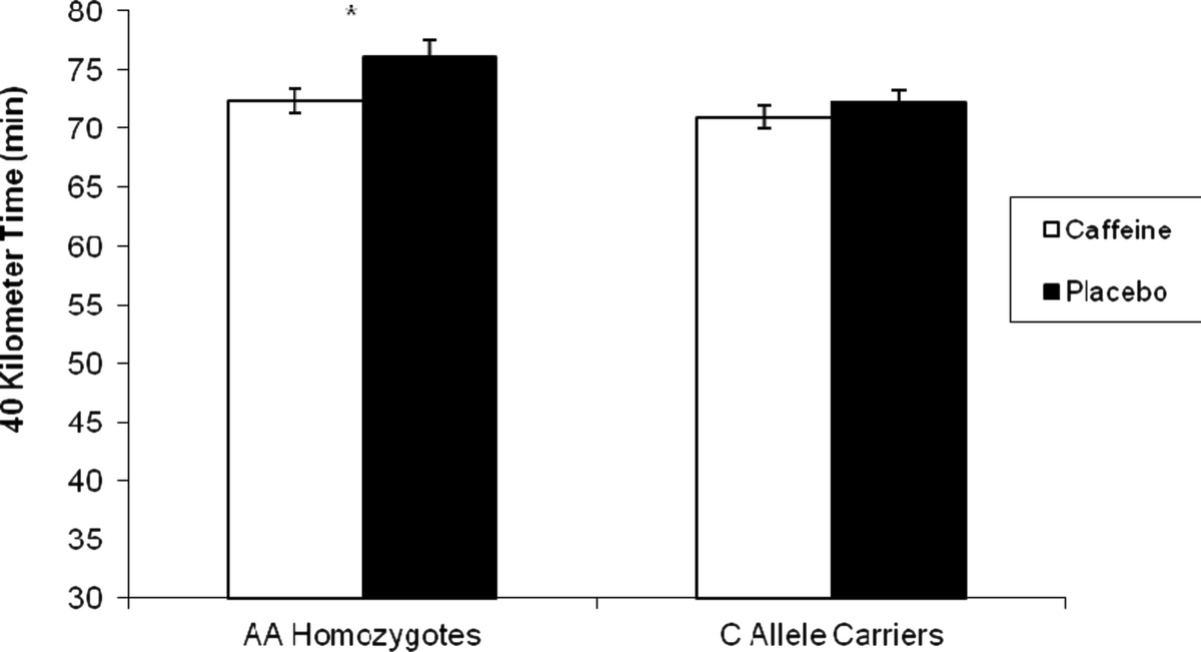
**Average (mean ± SE) 40 kilometer time for the caffeine and placebo treatments for both groups**. *-Significantly (*p *< 0.05) larger decrease in 40 K time than the C allele carriers.

**Figure 2:**
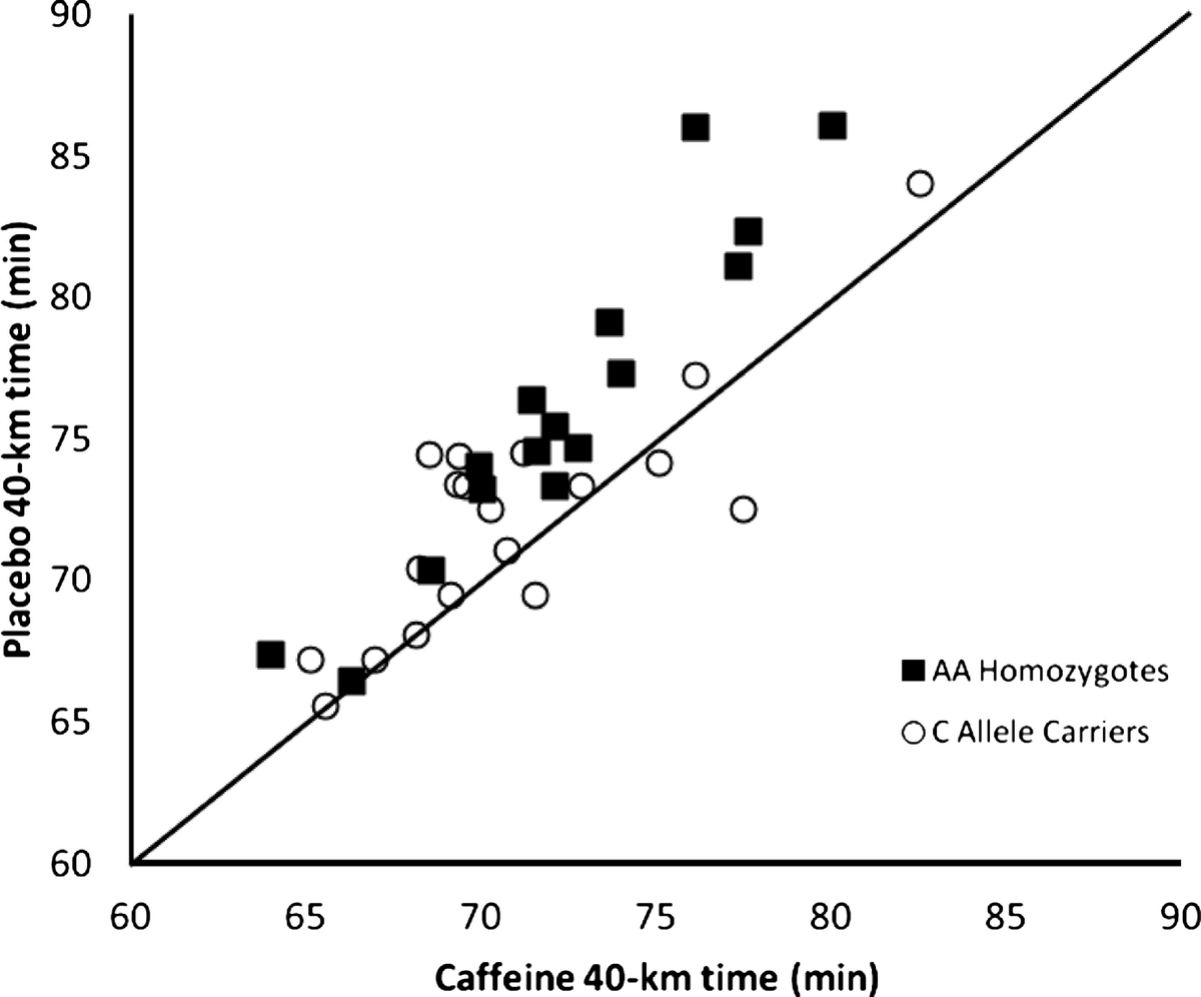
**40-km time in both the placebo condition (y-axis) and the caffeinated condition (x-axis) for both AA homozygotes and C allele carriers**. The line of identity is plotted and reflects no difference between the two trials. Data points above the line of identity reflect an improved 40-km time in the caffeinated condition.

Figure 1 displays the average 40-km times for both groups. There was a significant (*p *< 0.001) main effect for Treatment (Caffeine < Placebo) and a significant (*p *= 0.005) Treatment × Genotype interaction, such that caffeine lowered average (mean ± SD) 40-km time in AA homozygotes (4.9%; caffeine = 72.4 ± 4.2 min, placebo = 76.1 ± 5.8 min) to a greater degree than the C allele carriers (1.8%; caffeine = 70.9 ± 4.3 min, placebo = 72.2 ± 4.2 min). Caffeine significantly decreased 40-km time in the AA homozygotes (*p *< 0.001), with a strong trend towards decreased 40-km time in C allele carriers (*p *= 0.04). Individual data for the 40-km times in both groups are displayed in Figure 2. Note that data points above the line of identity reflect an improvement in 40-km time in the caffeine trial. Caffeine resulted in at least a 1-minute improvement in 40 k time in 15 out of the 16 AA homozygotes; whereas only 10 out of 19 C allele carriers observed this degree of improvement. Average RPE, VO_2_, RER and heart rate for the 40-km time trial are shown in Table 2. There was a main effect for Treatment for both VO_2 _and HR, with both variables higher in the caffeinated condition versus placebo (*p *< 0.001). Furthermore, there was a main effect of Genotype for VO_2_, with C allele carriers exhibiting significantly higher average VO_2 _than AA homozygotes (*p *= 0.03). There were no significant main effects or interaction effects for RPE or RER.

**Table 2:** Average (mean ± SD) values during the 40 k trial for Ratings of Perceived Exertion, VO_2_, Respiratory Exchange Ratio, and Heart Rate

RPE	Genotype	Caffeine	Placebo
	AA	14.3 ± 1.6	14.2 ± 1.6

	C	15.0 ± 1.4	14.9 ± 1.4

VO_2 _(L·min^-1^)^ab^			

	AA	3.08 ± 0.41	2.88 ± 0.49

	C	3.43 ± 0.48	3.23 ± 0.48

RER			

	AA	0.92 ± 0.05	0.91 ± 0.04

	C	0.94 ± 0.05	0.94 ± 0.04

HR (beats per min)^a^			

	AA	162 ± 10	153 ± 11

	C	170 ± 13	163 ± 14

^a^-Main effect for Treatment (*p *< 0.05)^b^-Main effect for Genotype (*p *< 0.05)

## Discussion

The major finding of the present study is that caffeine affects 40-kilometer time trial performance in cyclists homozygous for the A variant to a greater degree than those who possess the C variant. Specifically, caffeine decreased 40-km time by an average of 3.8 minutes in the AA homozygotes as compared to 1.3 minutes in the C allele carriers. To our knowledge, this is the first study to implicate a specific polymorphism as a potential cause of the variation in the ergogenic effect of caffeine supplementation.

Sachse et al. [10] observed slower caffeine metabolism in C allele carriers who smoke, suggesting that this CYP1A2 polymorphism may affect the inducibility of the Cytochrome P450 enzyme. Caffeine has also been shown to increase risk of heart disease in C allele carriers but not AA homozygotes [11,12], ostensibly because caffeine is metabolized at a higher rate in the AA homozygotes. Given these prior findings, it could be hypothesized that a slower metabolism would be advantageous for maximizing the ergogenic benefit of caffeine. Alternatively, Hallstrom et al. [13] found that coffee consumption was associated with decreased bone mineral density in AA homozygotes, but not C allele carriers. The authors speculated that the rapid accumulation of caffeine metabolites may have been responsible for this finding [13]. In support of this contention, paraxanthine and theophylline (downstream metabolites of caffeine metabolism) have higher binding affinities with adenosine receptors than caffeine [16]. Thus, it is possible that a faster caffeine metabolism in AA homozygotes created a more rapid production of paraxanthine and/or theophylline and therefore enhanced the ergogenic effect. This possibility is speculative as no markers of caffeine metabolism were available. Future studies should determine caffeine metabolism during exercise across these genotypes to better determine the mechanism of the observed effect.

Despite the fact that there was a significant Genotype × Treatment interaction for 40-km time, it should also be noted that the AA homozygotes had a slower placebo 40-km time and the caffeine supplementation served to decrease 40-km time for AA homozygotes to a level comparable to C allele carriers (Figure 1). This raises the concern that the results were driven by a difference in cycling performance capabilities between the two groups, rather than the genetic polymorphism. Collomp et al. [17] observed that caffeine improved swimming velocity in trained, but not untrained swimmers. O'Rourke et al. [18] observed a similar 5-km performance improvement from caffeine in both well-trained and recreational runners. Thus, one would expect performance capabilities to have no effect on caffeine response, or to affect it in the opposite direction of what was observed in the present study. Nonetheless, we further addressed the difference in performance between the two groups in a follow-up analysis. Because the higher 40-km time in AA homozygotes was primarily driven by four cyclists whose 40 k times during the placebo trial were greater than 80 minutes (see Figure 2), we removed these four subjects from the dataset for the follow-up analysis. This resulted in similar 40-km times in the placebo condition between the two groups, yet caffeine still had a significantly (*p *= 0.047) greater effect in AA homozygotes (caffeine = 70.5 ± 3.0 min, placebo = 73.5 ± 3.8 min) compared to the C allele carriers (caffeine = 70.9 ± 4.3 min, placebo = 72.2 ± 4.2 min). Caffeine resulted in at least a 1-minute improvement in 40 k time in all but one of the AA homozygotes; whereas only about half of C allele carriers responded to that extent (Figure 2). Thus, our data support the contention that it is the genetic polymorphism and not the performance capabilities of the respective groups that explain our observations.

Although data from the present study clearly suggest a potential role of this polymorphism in influencing the ergogenic response of caffeine in cyclists, care should be taken in extrapolating these findings. It is unknown if there is a similar genetic influence for other modes of exercise and/or for short-duration high-intensity exercise. Furthermore, we used trained cyclists in the present study and our findings cannot be extrapolated to sedentary individuals. Neither can it be suggested that this polymorphism is the only source of variation or even the only source of genetic variation involved. Finally, although we have outlined a potential mechanism that explains the current findings, it should be emphasized that the mechanistic causes of our findings cannot be determined from the present data. Future studies should determine whether these findings can be replicated using other modes of exercise and in other populations. Other candidate polymorphisms should also be identified and evaluated.

## Conclusions

In summary, data from the present study suggest that caffeine potentiates a larger ergogenic effect for cycling performance in individuals homozygous for the A variant of the studied CYP1A2 polymorphism. The mechanism(s) of this selective ergogenic effect are unknown and future studies should seek to establish the impact of this polymorphism on caffeine metabolism during exercise. While these findings elucidate a possible source of variance in the ergogenic effect of caffeine, other factors, including other genetic polymorphisms, may also influence caffeine responses during exercise.

## Competing interests

The authors declare that they have no competing interests.

## Authors' contributions

CJW planned the study, assisted with data collection and wrote the bulk of the manuscript. MJS helped with study design, data interpretation and manuscript preparation. MKB helped with study design, performed genotyping and manuscript preparation. DJB helped with study design and data collection. MM helped with data collection and manuscript preparation. NDL assisted with data collection, study design and manuscript preparation. Both WD and MH performed genotyping and manuscript preparation. All authors read and approved the final manuscript.
