# Erratum to: The influence of a CYP1A2 polymorphism on the ergogenic effects of caffeine

**DOI:** 10.1186/s12970-015-0079-6

**Published:** 2015-05-30

**Authors:** Christopher J Womack, Michael J Saunders, Marta K Bechtel, David J Bolton, Michael Martin, Nicholas D Luden, Wade Dunham, Melyssa Hancock

**Affiliations:** Department of Kinesiology, Human Performance Laboratory, James Madison University, 261 Bluestone Drive, MSC 230, Harrisonburg, VA 22801 USA; Department of Biology, James Madison University, Harrisonburg, VA 22801 USA

## Correction

Following publication of this article [1], we recently noticed that two subjects in this study were not assigned to the correct genetic groups. One of the AA homozygotes was incorrectly entered as a C allele carrier and we had the opposite situation for a second subject.

We have re-run the primary statistical analysis with the subjects assigned to their correct groups. The corrected analysis produced very similar results to our initial, published findings as we observed a significant treatment effect and a significant genotype x treatment interaction due to a higher degree of response in the AA homozygotes.

Correcting our dataset did result in very slight differences in the mean values for 40 k time. The changes are presented in Table 1.

**Table 1:** .

**Genotype**	**Placebo**	**Caffeine**
**A. Original Data: 40-km cycling time for Placebo and Caffeine conditions for AA homozygotes and C-allele carriers**		
AA	76.1 +/- 5.8 min	72.4 +/- 4.2 min
C	72.2 +/- 4.2 min	70.4 +/- 4.3 min
**B. Corrected Data: 40-km cycling time for Placebo and Caffeine conditions for AA homozygotes and C-allele carriers**		
AA	75.1 +/- 6.1 min	71.6 +/- 4.3 min
C	73.1 +/- 4.5 min	71.6 +/- 4.4 min
